# Reduction of heart rate by omega-3 fatty acids and the potential underlying mechanisms

**DOI:** 10.3389/fphys.2012.00416

**Published:** 2012-10-30

**Authors:** Jing X. Kang

**Affiliations:** Laboratory for Lipid Medicine and Technology, Massachusetts General Hospital and Harvard Medical SchoolBoston, MA, USA

**Keywords:** omega-3 fatty acids, cardiac sudden death, heart rate, membrane electrical excitability, ion channel inhibition

## Abstract

An elevated resting heart rate is one of the strongest predictors of cardiovascular mortality and is independently associated with sudden cardiac death (SCD). Agents capable of reducing heart rate without significant side effects are therefore of particular interest for the prevention of SCD. Recent human and animal studies have shown that omega-3 fatty acids can reduce heart rate. Our work has shown that omega-3 fatty acids significantly reduce membrane electrical excitability of the cardiac myocyte by lowering its resting membrane potential and the duration of the refractory period through inhibition of ion channels. We propose that these actions may be the underlying mechanisms for the omega-3 fatty acid-induced reduction of heart rate observed in both humans and animals. The heart rate-lowering capability of omega-3 fatty acids may contribute to their preventive effect against SCD.

## Introduction

The cardioprotective effects of omega-3 fatty acids have become widely recognized. One of the most significant effects is the prevention of sudden cardiac death (SCD) (de Lorgeril et al., 1994; GISSI-Prevenzione, 1999; Marchioli et al., 2002; Leaf et al., 2005, 2003), which is generally defined as death within 1 h of the onset of symptoms, and is most frequently caused by ventricular fibrillation. Although the underlying mechanisms for this preventive effect are not yet well understood, the reduction of heart rate by omega-3 fatty acids may be an important factor contributing to decreased risk for SCD.

An elevated resting heart rate is one of the strongest predictors of cardiovascular mortality. In particular, a resting heart rate of >70–90 beats per minute (bpm) is independently associated with SCD. Multiple prospective studies have shown that even after adjusting for common cardiovascular health-related variables, such as age, weight, smoking, alcohol consumption, diabetes, blood pressure, physical activity, blood cholesterol, medications, and socioeconomic status, elevated heart rate remains a risk factor for SCD and a predictor of time to cardiac death (Shaper et al., 1993; Palatini et al., 1999). In the Framingham cohort, cardiovascular and coronary mortality rates increased with progressively higher resting heart rates irrespective of age or sex, although the fraction of SCD rose sharply in men 35–65 years old (Kannel et al., 1987). In general, similar relationships between heart rate and cardiovascular death exist in men and women, but the association is weaker in women (Kannel et al., 1987; Palatini et al., 1999). Interestingly, heart rate is predictive of SCD in men both with and without a history of ischemic heart disease (IHD) (Wannamethee et al., 1995), and in some cases this relationship is even stronger in men without pre-existing IHD (Shaper et al., 1993).

Given these consistent and robust findings, drugs or supplements that reduce heart rate are of particular interest for preventing SCD. In fact, studies that have examined outcomes for patients with chronic heart failure have revealed that pharmacotherapy to reduce heart rate is associated with better outcomes for at least 5 years (Franke et al., 2012). Beta-adrenergic blockers, cardiotonic agents such as ivabradine, and ACE inhibitors have been prescribed for the purpose of lowering heart rate and reducing mortality, though they may present adverse effects (Arshad et al., 2008; Böhm et al., 2012). Thus, agents that are able to reduce heart rate without significant side effects may be valuable for the prevention of SCD. Omega-3 fatty acids, a class of essential nutrients primarily found in fish oil consisting of eicosapentaenoic acid (EPA) and docosahexaenoic acid (DHA), have been consistently shown to lower heart rate (Grimsgaard et al., 1998).

The relationship between omega-3 fatty acids and cardiovascular disease is well studied, and has appeared inconsistent at times (Harris et al., 2006; Kromhout et al., 2010; Rizos et al., 2012). Still, it is important to consider that there is strong mechanistic evidence supporting a protective effect of omega-3 fatty acids on cardiovascular disease. In this review, I will provide an overview of the evidence for the heart rate-lowering effects of omega-3 fatty acids both in animals and humans, and explain how findings from our *in vitro* work provide a likely mechanism by which omega-3 fatty acids act on cardiac myocytes to reduce heart rate.

## Reduction of heart rate by omega-3 fatty acids

The effect of omega-3 fatty acids on heart rate has been observed in many different populations, both with and without cardiovascular disease. A meta-analysis of 30 randomized, double-blind, placebo-controlled trials concluded that fish oil consumption can significantly reduce heart rate (Mozaffarian et al., 2005). In particular, the effect was greater in people whose baseline heart rate was higher: in the overall pooled estimate, fish oil decreased heart rate by 1.6 bpm compared to placebo, but reduced heart rate by 2.5 bpm in trials with a median baseline heart rate of ≥ 69 bpm. Furthermore, the ability of fish oil to reduce heart rate appeared to depend on the length of treatment. When a trial lasted for more than 12 weeks, fish oil reduced heart rate by 2.5 bpm. However, when the trial lasted for less than 12 weeks, fish oil had little effect on heart rate. Interestingly, this meta-analysis also confirmed that heart rate reduction did not vary significantly by fish oil dose (Mozaffarian et al., 2005). Furthermore, another randomized, controlled trial on 18 men with a history of myocardial infarction and ejection fractions of <40% showed that those given omega-3 fatty acids experienced a 5 bpm reduction in resting heart rate and an improved 1-min heart rate recovery after exercise (O'Keefe et al., 2006).

In addition, several large-scale, population-based studies showed that increased dietary fish and omega-3 fatty acid intake was associated with a significant reduction in heart rate. Dallongeville et al. (2003) analyzed 2 years of data on 9758 men without coronary heart disease from France and Ireland, grouping the men into four statistical categories based on how much fish they consumed per week (less than once, once, twice, and more than twice/week). They found that heart rate decreased across the categories of fish intake and was lower in fish consumers than in non-consumers, even after adjustments for age, location, level of education, physical activity, smoking habits, alcohol consumption, body mass index, and antiarrhythmic medications (Dallongeville et al., 2003). Studies by Mozaffarian and colleagues further examined the associations between fish intake and a variety of cardiac measures (Mozaffarian et al., 2006a,b). Their results showed that high fish consumption is associated with a heart rate reduction of approximately 3.2 bpm. They also found that an estimated 1 g/day higher EPA + DHA intake was associated with a heart rate reduction of 2.3 bpm. Functionally, this improvement in heart rate (−3.2 bpm) corresponds to a ~7.5% lower risk of SCD (Mozaffarian et al., 2006b).

Fish oil also effectively reduces heart rate during times of increased cardiac demand such as exercise. A study of 25 Australian football players revealed that 6g/day of fish oil reduced heart rate during submaximal exercise over a period of 5 weeks (Buckley et al., 2009). Likewise, another randomized, placebo-controlled study of 16 exceptionally fit male cyclists taking 8g/day of fish oil for 8 weeks also found a reduction in heart rate during exercise. Heart rate during incremental workloads to exhaustion was lowered, as was peak heart rate, oxygen consumption, and heart rate during steady submaximal exercise (Peoples et al., 2008). However, decreased heart rate from fish oil during exercise is not contingent on physical fitness; in a study of 65 sedentary, overweight volunteers who consumed tuna fish oil for 12 weeks, resting heart rate and heart rate response to submaximal exercise were decreased (Ninio et al., 2008). Thus, fish oil reduced heart rate both at rest and during the stress of exercise, irrespective of the relative fitness level of the participant.

Another set of interesting findings comes from a population perhaps the least likely to experience cardiovascular illness: infants. Term infants treated with varying amounts of DHA in their formulas for 12 months show that DHA supplementation reduces heart rate compared to infants whose formula does not contain DHA, with no evidence of a dose response (Pivik et al., 2009; Colombo et al., 2011). These data are noteworthy in that they reinforce the non-specific impact of fish oil on heart rate, and suggest that almost any cohort may benefit from fish oil in this manner.

Finally, Harris and colleagues performed a small prospective study that provides a valuable indication of which mechanisms are likely to underlie the omega-3 fatty acid-driven reduction in heart rate. The group enrolled heart transplant patients, ensuring that their transplants had occurred more than three months prior and there had been no transplant-related hospitalizations (Harris et al., 2006). The revealing aspect of this study is that transplanted hearts are functionally denervated of the vagal nerve, and thus devoid of sympathetic and parasympathetic inputs. The patients were randomly assigned to receive either a corn oil placebo or EPA/DHA for 4–6 months, and at the end of the study the patients in the omega-3 fatty acid group had heart rates on average 5.4 bpm lower than baseline, whereas the corn oil group showed no change (Harris et al., 2006). These findings suggest that omega-3 fatty acids impact heart rate at the level of the myocardium itself, and are in particular consistent with the idea that the voltage-gated ion channels that control the pacemaker currents in the heart are influenced by omega-3 fatty acids.

Similar reductions in heart rate due to omega-3 fatty acids have been observed in animals. In a rat model, animals fed a DHA-enriched diet had lower heart rates than animals fed a control diet, a pattern that was achieved by 2 months and maintained until the end of the 32-week study (Ayalew-Pervanchon et al., 2007). In a hyperinsulinemic model, rats fed a diet containing DHA showed lower heart rates and a shortened QT interval as compared to rats fed and EPA-rich diet (Rousseau et al., 2003). Similarly, in rabbits fed a diet enriched with 2.5% (w/w) fish oil for three weeks, sinus cycle length and heart rate were reduced compared to animals fed a 2.5% oleic sunflower oil for the same period of time (Verkerk et al., 2009). A series of studies by Billman and colleagues has also provided important information about omega-3 fatty acids and heart function. Omega-3 fatty acid infusions into 13 intact, conscious, exercising dogs highly susceptible to ischemia-induced ventricular fibrillation were able to prevent ventricular fibrillation in 10 of the 13 dogs tested. The antiarrhythmic effect was associated with slowing of the heart rate, shortening of the QT interval, reduction of the left ventricular systolic pressure, and prolongation of the electrocardiographic atrial-ventricular conduction time (PR interval) (Billman et al., 1997). Additional work showed that dietary omega-3 supplementation reduces resting heart rate and increases heart rate variability in dogs with previous but healed myocardial infarction, as well as in dogs that are either susceptible or resistant to ventricular fibrillation (Billman and Harris, 2011; Billman, 2012). Moreover, these reductions in baseline heart rate were maintained during challenges (i.e., exercise or acute myocardial ischemia), but omega-3 did not alter the amount of change induced by challenge; these findings are consistent with the idea that omega-3 supplementation impacts intrinsic heart rate rather than autonomic regulation of the heart. Interestingly, there is also evidence that under certain conditions omega-3 may actually be pro-arrhythmic by reducing myocyte excitability during acute regional ischemia (Coronel et al., 2007).

Overall, it is apparent that a wide range of human and animal subjects, with or without cardiac disease, all respond to omega-3 fatty acid supplementation with reductions in resting and stress-induced heart rates. These findings suggest a highly consistent and robust effect of omega-3 fatty acids on heart rate.

## Potential mechanisms underlying the heart rate-lowering effect of omega-3 fatty acids

**Figure 1:**
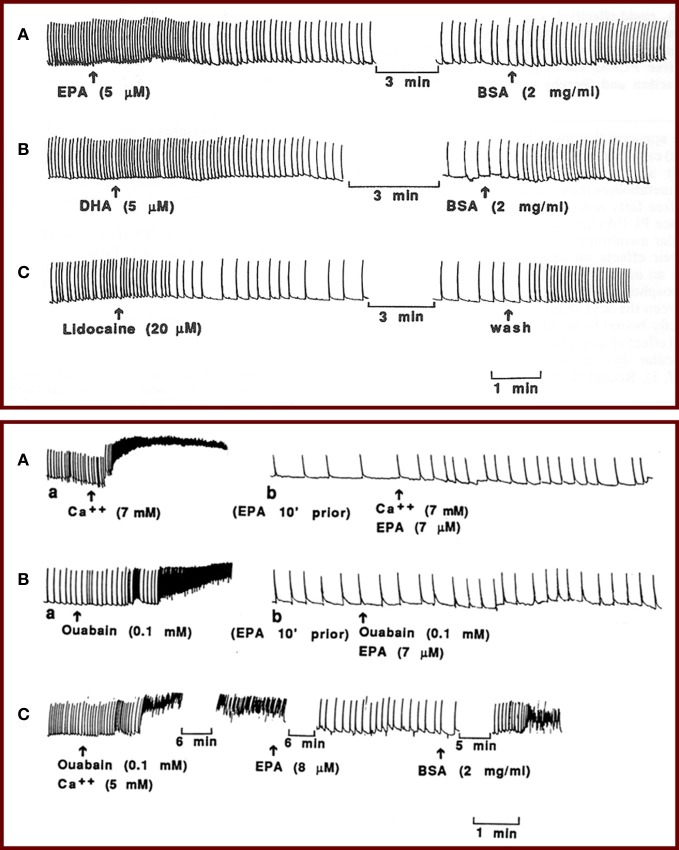
**(Top panel)** Effects of EPA and DHA on the contraction of isolated neonatal rat cardiomyocytes. Perfusion of the myocytes with 5 μM EPA **(A)** or 5 μM DHA **(B)** reduced the beating rate by 50% within 2 min, and addition of BSA to the perfusion solution quickly reversed the effect. Tracing **(C)** shows a similar effect of lidocaine (20 μM) on the contraction of the cardiac myocytes. **(Bottom panel)** Tracings show prevention and termination of arrhythmia by EPA. Perfusion of the myocytes with a solution containing 7 AM Ca^2+^ (**A**, a) or 0.1 mM ouabain (**B**, a) induced contracture and fibrillation before perfusion with EPA. Washing the cells with medium (Ca^2+^ = 1.2 mM) returned the fibrillations to the original beating rate (not shown). Then the cells were perfused with medium containing 7 μM EPA. After 5–8 min, when the beating rate was slowed, addition of 7 mM Ca^2+^ (**A**, b) or 0.1 mM ouabain (**B**, b) failed to induce contracture or fibrillation in the same cells. The slow beating rates were subsequently returned to the original rates by perfusion with BSA (not shown). **(C)** Alternatively, after induction of fibrillation by ouabain (0.1 mM) plus Ca^2+^ (5 mM), addition of IEPA (8 μM) terminated the fibrillation and led to slow beating, and subsequent addition of BSA (2 mg/ml), still in the presence of ouabain and high external Ca^2+^ concentration, reinstated fibrillation.

Although omega-3 fatty acids may reduce heart rate through several different mechanisms, our early studies demonstrated a direct effect of omega-3 fatty acids on cardiac cell membrane electrical excitability that contributes to reduced heart rate. We used isolated, neonatal rat cardiac myocytes that retain spontaneous beating behavior, allowing us to assess the effect of EPA and DHA on contraction as well as electrophysiological activity without neural or hormonal input. We found that EPA and DHA promptly reduced the contraction rate of the cardiac myocytes by 50–80% without a significant change in the amplitude of the contractions. This effect of omega-3 fatty acids on the excitability of the cells was similar to that produced by the class I antiarrhythmic drug lidocaine (Figure 1, top) (Kang and Leaf, 1994). In addition, we showed that EPA and DHA can prevent as well as terminate fibrillation, characterized by chaotic, asynchronous beating and contractures, induced either by high extracellular calcium concentrations and/or ouabain (Figure 1, bottom) (Kang and Leaf, 1994). Inhibitors of fatty acid metabolism, however, have no effect on omega-3 fatty acid-induced heart cell contraction, indicating that the free fatty acids do not need to be metabolized into byproducts to cause a reduction in heart rate (Kang and Leaf, 1994). The omega-3 fatty acid-induced reduction in the beating rate could be readily reversed by cell perfusion with fatty acid-free bovine serum albumin, indicating that omega-3 fatty acids likely do not need to be incorporated into the membrane phospholipid or covalently linked to membrane components in order to be effective. These results suggest that omega-3 fatty acids in their free form can suppress the automaticity of cardiac contraction and thereby exert their heart rate-lowering effects.

**Figure 2:**
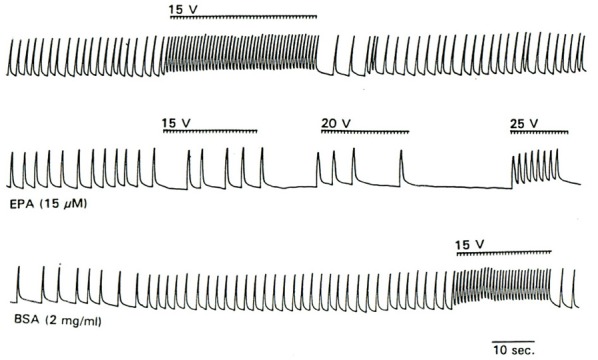
**Effect of EPA on the response of cultured myocytes to electrical pacing.** Trace shows the response of cell contraction to the electrical stimuli (as indicated by the bars above the contraction trace) before perfusion with EPA (top trace), during perfusion with 15 μM EPA (middle trace) and after washout with BSA.

The reduction of electrical excitability of cardiac myocytes by omega-3 fatty acids can be demonstrated directly by their response to electrical pacing (Kang and Leaf, 1996). As shown in Figure 2, prior to addition of EPA to the cells, application of a series of stimulating impulses elicited a rapid beating synchronized with the impulse rate. 3–5 min after perfusion of the cells with 15 uM EPA, when a slowing of the beating rate had occurred, application of the same (15 V) or even stronger electrical stimuli failed to boost the beating rate. When the cells were washed with medium containing delipidated BSA (2 mg/ml) for 2–3 min, stimulation of the cells with 15 V field strength induced a response similar to that observed prior to addition of EPA (Figure 2) (Kang and Leaf, 1996). These results further suggest that omega-3 fatty acids have an inhibitory effect on the electrical automaticity/excitability of the cardiac myocytes.

**Figure 3:**
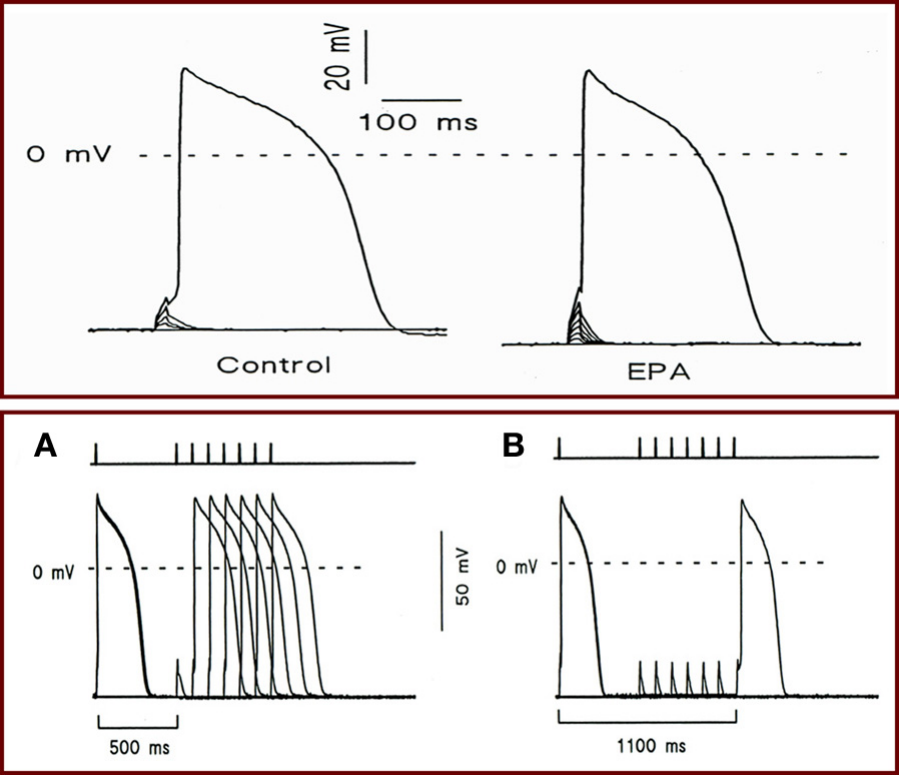
**(Top panel)** Effect of EPA on holding voltage at constant imposed current, threshold voltage, and current required to attain action-potential threshold. Holding potential was initially set at −70 mV, and cells were stimulated with a series of depolarization pulses in 3- or 4-pA increment at 10 s intervals. Recordings showing the holding transmembrane potentials, the threshold potentials, and the currents required to elicit an action potential in a cell before (Left) and after (Right) exposure to 10 μM EPA. **(Bottom panel)** The effect of EPA on the cycle length of excitability. Cells were given a pair of superthreshold electrical stimuli at 0.1 Hz (necessary to elicit action potential in the presence of EPA) with various intervals from 500 to 1500 ms in 100 ms increments. Bars at top indicate the time intervals between two stimuli. **(A)** Recording showing the cycle length (600 ms) before 10 μM EPA exposure. **(B)** Recording showing cycle length (1100 ms) 3 min after EPA exposure.

To better elucidate the mechanism of action of omega-3 fatty acids on heart rate, we employed a patch-clamp technique to examine the electrophysiological activity in isolated neonatal rat cardiac myocytes. First, we induced the action potential in the myocytes exposed to EPA or DHA and measured the strength of the current required to elicit an action potential (Kang et al., 1995). We found that EPA increased the strength of the depolarizing current needed to provoke an action potential and lengthened the cycle of excitability. These changes were due to an increase in the threshold for action potential and a more negative resting membrane potential. There was a progressive prolongation of intervals between spontaneous action potentials and a slowed rate of phase 4 depolarization (Figure 3) (Kang et al., 1995). These results demonstrate that omega-3 fatty acids can indeed reduce membrane electrical excitability and provide an electrophysiological basis for the heart rate-lowering effects of free omega-3 fatty acids. These findings are consistent with the observations that omega-3 fatty acids can profoundly reduce the contraction rate of cardiac myocytes (Kang and Leaf, 1994).

**Figure 4:**
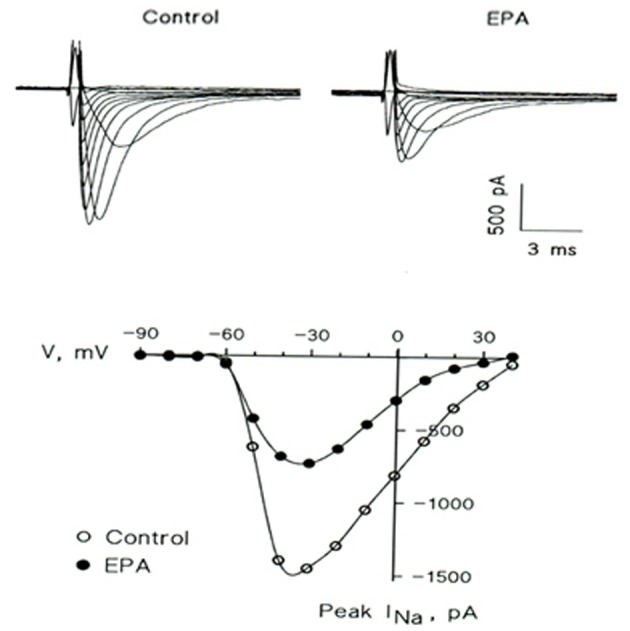
**EPA suppresses the voltage-activated Na+ current (*I*_Na_) in a representative cultured neonatal rat ventricular myocyte.** (Upper) Superimposed traces in the absence (Control) and presence (EPA) of 5 μM EPA. The currents were elicited by 10 mV increment voltage steps (20 ms; 0.2 Hz) from a holding potential of −80 mV down to −90 mV and up to 40 mV. (Lower) Current-voltage relations of peak *I*_Na_ in the absence (◦) and presence (•) of 5 μM EPA.

At this point in our research, the manner by which omega-3 fatty acids reduce membrane excitability was still unclear. Therefore, we tested the effects of omega-3 fatty acids on single ion channel activity in neonatal rat cardiac myocytes. The results demonstrated a prompt inhibitory action of omega-3 fatty acids on the Na+ currents through fast sodium channels responsible for the phase 0 of the action potential in isolated neonatal rat cardiac myocytes. The inhibition of this ion channel was dose, time, and voltage dependent, but not use dependent (Figure 4) (Xiao et al., 1995). Subsequent studies have demonstrated that other ion channels, such as calcium channels, can also be affected by omega-3 fatty acids to varying degrees (Leaf, 2001). These findings provided the ionic basis for the marked electrophysiological effects of omega-3 fatty acids on myocytes, and explained why omega-3 fatty acids are capable of reducing heart rate.

## Conclusions

Regulation of heart rate in humans is highly complex. Sympathetic output, vagal tone, and systolic and diastolic left ventricular function are only a few of the factors that contribute to the regulation of heart rate. While omega-3 fatty acids could potentially affect any or all of these factors, our studies strongly suggest a direct impact of omega-3 fatty acids (Leaf et al., 1999a,b). Our cellular work has shown that omega-3 fatty acids significantly reduce membrane electrical excitability of the cardiac myocyte by lowering its resting membrane potential and the duration of the refractory period through inhibition of ion channels. We propose that these actions may be the underlying mechanisms for the omega-3 fatty acid-induced reduction of heart rate observed in both humans and animals. Given the close relationship between heart rate and SCD, the direct impact of omega-3 fatty acids on the cardiac membrane to reduce heart rate may be a critical determinant of the preventive effect of omega-3 fatty acids against SCD. Thus, increasing intake of omega-3 fatty acids would likely benefit individuals at risk for SCD.

### Conflict of interest statement

The author declares that the research was conducted in the absence of any commercial or financial relationships that could be construed as a potential conflict of interest.
